# The Impact of Social Media and Infection Perception on the Intentions of Recreational Vehicle Tours: An Extended Model of Goal-Directed Behavior

**DOI:** 10.3390/bs14110986

**Published:** 2024-10-24

**Authors:** Chao Zeng, Zihan Yang, Yufan Zhai, Li Yu

**Affiliations:** 1 College of Traffic and Transportation, Chongqing Jiaotong University, Chongqing 400074, China; zengchao0602@cqjtu.edu.cn (C.Z.); zhaiyufan1227@mails.cqjtu.edu.cn (Y.Z.); 2 School of Geography and Planning, Cardiff University, Cardiff CF10 3AT, UK; yul@cardiff.ac.uk

**Keywords:** RV tourism, tourism intentions, model of goal-directed behavior (MGB), structural equation model (SEM), social media, perception of infection

## Abstract

Despite the rapid expansion of China’s RV (recreational vehicle) tourism industry, research on RV tourist behavior remains limited. This study develops an extended model of goal-directed behavior (EMGB) that more comprehensively explains the formation of tourists’ intentions to engage in RV tourism. The EMGB incorporates critical factors, including the perception of infection (PI), social media influence (SM), infrastructure perception (IP), and perceived advantages of drive tourism (PAD), into the original goal-directed behavior model (MGB). Results from a survey involving 545 RV tourists reveal that the EMGB achieves a satisfactory fit with the data and explains a larger proportion of variance in behavioral intentions. Notably, all variables within the original MGB remain significant predictors of both desire and intention. Furthermore, this study identifies factors such as Time and Economy capability, age, and region as significant moderators of the decision-making process for RV tourists. These findings offer valuable insights to marketers and governmental bodies, empowering them to make more reasonable decisions and devise strategies that cater to the diverse needs and preferences of RV tourists in the future.

## 1. Introduction

Since the early 20th century, RV tourism has emerged as a unique form of travel, demonstrating robust vitality globally and becoming a highlight in the tourism markets of developed countries such as those in Europe and the United States [1,2]. With the deepening implementation of China’s transportation and tourism integration policies, along with the continuous improvement in RV tourism industry policies and regulations, the RV tourism industry of China has also entered a rapid development trajectory. According to the 2023 Transportation Industry Development Statistical Report, China’s total road length exceeds 5.44 million km, ranking among the highest in the world, providing a solid infrastructure foundation for the development of RV tourism. The number of RVs in China has been steadily increasing, reaching 213,400 units by the end of 2023, making China a significant player in the global RV market [3]. To further promote the development of the RV tourism industry, understanding tourist choice behaviors and the influencing factors of destination selection is crucial for the planning and marketing of tourist destinations.

In exploring the development path of the RV tourism industry, a thorough analysis of tourist choice behavior and its underlying influencing factors is of significant value for formulating effective marketing strategies. The tourism industry often faces severe impacts due to various unexpected events such as diseases, epidemics, seasonal flu, and global pandemics. These include major crises like the global financial crises in 1997 and 2008, the SARS epidemic in 2003, and various social upheavals and earthquakes [4,5,6,7]. RV tourism, which is defined as travel in recreational vehicles equipped with sleeping, cooking, and sanitary facilities that provide both transportation and living accommodations, is a subset of drive tourism, as it inherently involves the use of personal vehicles for self-guided travel to various destinations [8]. This form of tourism has gained popularity during the COVID-19 pandemic because of its unique privacy and ability to avoid crowds [9]. This trend suggests that changes in drive tourism behavior during pandemics can serve as a reference model for future annual susceptible seasons [9,10]. It is crucial to study shifts in traveler decision-making mechanisms during such periods to understand consumer psychology and develop adaptive marketing strategies and measures that meet the specific market demands of these times.

Tourists enjoy sharing their memorable experiences on social media. This sharing behavior may spark tourists’ future travel intentions and attract more tourists to participate in RV tourism [11,12]. It was noted that content related to drive tourism surged on social media during the COVID-19 pandemic. Therefore, this study considers the impact of related content on social media platforms on tourists’ choices of RV tourism during viral outbreaks. Consequently, it is proposed that further investigation is needed into the complex interplay between perceived infection risk and social media in the decision-making process for RV tourism.

Currently, research on the decision-making process for RV tourism, particularly regarding the combined effects of infection risk and social media factors, is insufficient. Therefore, this study aims to analyze the RV tourism decision-making process under their joint influence, providing constructive recommendations for the development of the RV tourism industry during susceptible seasons. As a significant branch of drive tourism [13], RV tourism should be influenced by the overall environment of drive tourism. Therefore, this study innovatively proposes that social media and perception of infection may shape the perceived advantages of drive tourism, thereby influencing tourists’ desire and intention to engage in RV tourism.

A comprehensive review of the existing literature reveals that social psychology theories are widely used in the study of tourism behavior intentions [14,15], yet current research has not utilized these theories to analyze the decision-making process of RV tourism behavior. Therefore, this study constructs and validates an extended model based on the model of goal-directed behavior (MGB). To analyze the behavior decision-making process of RV tourists more comprehensively, infrastructure perception, recognized as a critical factor in RV tourism, is integrated alongside social media, perceived infection risk, and perceived advantages of drive tourism. Understanding the role and mechanisms of these variables in the decision-making process of RV tourists is essential. Additionally, as multi-group invariance analysis is widely used to verify the stability and generalizability of path models across different sample groups [16] and to analyze differences shown in cross-group comparisons, this method is employed in this study. This approach delves into the differences in the RV tourism decision-making process among different groups, providing scientific evidence for market segmentation, target group positioning, and the formulation of personalized marketing strategies.

The findings of this study not only advance the theoretical understanding of RV tourism by expanding the original MGB but also enhance the understanding of the roles of perceived infection risk and social media in the decision-making process of RV tourists during susceptible seasons. Furthermore, this study explores the decision-making process differences among various groups of RV tourists. These insights will help stakeholders in the RV tourism industry better understand potential tourists, enabling them to make informed decisions to serve RV tourists and promote the development of the RV tourism industry.

## 2. Literature

### 2.1. RV Tourism and Tourists’ Behavioral Intentions

RV tourism and camping have gained popularity in North America, Australia, Europe, and, more recently, in some emerging economies [17,18]. However, unlike camping, RV tourism constitutes a form of travel where tourists engage in holiday activities while traveling in recreational vehicles (RVs), considering these vehicles as their primary accommodation option [19]. In recent years, there has been a growing preference for RV tourism as the RV industry has become an integral part of leisure tourism [20]. Not only that, but the flexibility of RVs provides tours with broader choices in destinations or travel routes [21]. RVs have undergone significant changes in size, width, and amenities over the past 100 years, offering refined comfort as a mode of transportation typically equipped with amenities like flat-screen TVs, microwaves, and air conditioning [22]. As articulated by Lumsdon and Page, the transportation aspect itself is central to the entire experience of this form of tourism [23]. Pearce and Wu suggested that RV tourism offers several advantages over other forms of drive tourism, such as its flexibility, which not only allows for journeys without strict time schedules but also permits users to carry more belongings, even pets, ensuring a more comfortable travel experience [19].

RV tourism holds a significant market share within the tourism industry, and the travel behavioral intentions of RV tourists have considerable research value. Therefore, it is essential to conduct further investigative research into the reasons why individuals engage in RV tourism [8,19]. However, academic studies in related fields mostly concentrate on other branches of the tourism industry, leaving relatively limited content on research regarding the behavioral intentions of RV tourism.

### 2.2. Theoretical Basis: The Model of Goal-Directed Behavior (MGB)

Research on travel behavioral intentions often utilizes social psychology theories to study the individual decision-making process [14,15,24]. In the realm of social psychology theories investigating individual decision-making processes, it is generally recognized that an individual’s attitude plays a significant role in their behavior, forming a research structure commonly known as the “attitude–behavior” relationship [25]. The theories primarily employed in studying travel behavioral intentions include the Theory of Reasoned Action (TRA), the Theory of Planned Behavior (TPB), and the model of goal-directed behavior (MGB) [26]. In 1980, Fishbein and Ajzen introduced the Theory of Reasoned Action (TRA), proposing that an individual’s actual behavior is determined by their behavioral intention (BI). In turn, behavioral intention is influenced by an individual’s attitude (AT) and subjective norm (SN) [27]. However, the TRA assumes that individual behavior is entirely under volitional control, neglecting the varying impacts of other relevant factors that may inhibit an individual to different extents. This limitation restricts the practical utility of the theory. In response to these limitations, Ajzen (1985) introduced the concept of Perceived Behavioral Control (PBC) into the Theory of Reasoned Action, expanding the model to become the Theory of Planned Behavior (TPB) [27]. Similar to the Theory of Reasoned Action, TPB considers an individual’s behavioral intention as the ultimate determinant of behavior, forming the core of both theories. The Theory of Planned Behavior has found extensive application across various disciplines and has been successfully validated in related research [28,29].

Although the Theory of Planned Behavior was refined by incorporating the concept of Perceived Behavioral Control, it still exhibits notable limitations in terms of emotions and motivations [26]. Conner and Armitage (1998) pointed out that integrating emotional and habitual processes into social psychological models can better understand human behavior [30]. Perugini and Bagozzi divided anticipated emotional variables into the following dimensions: positive anticipated emotion (PAE) and negative anticipated emotion (NAE), introducing them and Frequency of Past Behavior (FPB) into the Theory of Planned Behavior. Simultaneously, they included desire (DE) as a mediating variable, constructing the goal-directed behavior model, and they suggested that incorporating behavioral desire could effectively integrate and optimize attitudes, subjective norms, and perceived behavioral control, leading to more accurate predictions and judgments of individual behavior [26].

Revising existing social psychology theories can predict the behaviors of individuals in specific situations [26]. Taylor (2007) demonstrated the inclusion of specific additional variables to extend the MGB model to explain differences in intentions and behavior in specific contexts [31]. Bui and Kiatkawsin (2020) integrated social networking media into the goal-directed behavior model in their study to investigate the tourism intentions of Vietnamese adventure tourists [32]. Lee et al. (2012) added non-pharmaceutical interventions and H1N1 perception to better explore the mechanism of the H1N1 pandemic’s impact on the behavioral intentions of outbound tourists [24]. Meng and Choi (2016) integrated the perception of authenticity into their research and investigated its influence on the intentions of slow tourists [15]. Chen, Wang (2022) incorporated the variable of risk perception into their research to explore the Influence Mechanism of Intention to Proximity Travel under the COVID-19 pandemic [33].

### 2.3. The Behavioral Intention of RV Tourism and MGB

#### 2.3.1. Attitude, Subjective Norm, Perceived Behavioral Control, and Desire

The Theory of Reasoned Action (TRA), proposed by Martin Fishbein and Icek Ajzen (1980), posits that human behavior is determined by subjective evaluations of attitudes towards the behavior and subjective norms [34]. Attitude is a subjective evaluation of the approval level of an individual towards a behavior, while the subjective norm is the perceived social pressure, i.e., others’ opinions regarding the behavior [26]. According to TRA, individuals are more likely to engage in a behavior if they hold a positive attitude towards it and perceive positive social norms from others regarding that behavior [27,34]. The Theory of Planned Behavior (TPB), introduced by Martin Fishbein and Icek Ajzen (1985), integrates perceived behavioral control (PBC) into the TRA framework [27]. Perceived behavioral control refers to an individual’s subjective perception of their ability or degree of control in performing a behavior. It involves subjective judgments regarding the resources, skills, obstacles, support, and conditions necessary to enact a behavior, which significantly influences the formation of behavioral intentions [26,35]. Perugini and Bagozzi argued that as antecedents, attitudes, subjective norms, perceived behavioral control, and anticipated emotions do not directly impact individual behavioral intentions but rather influence them indirectly through desires [26].

**Figure 1:**
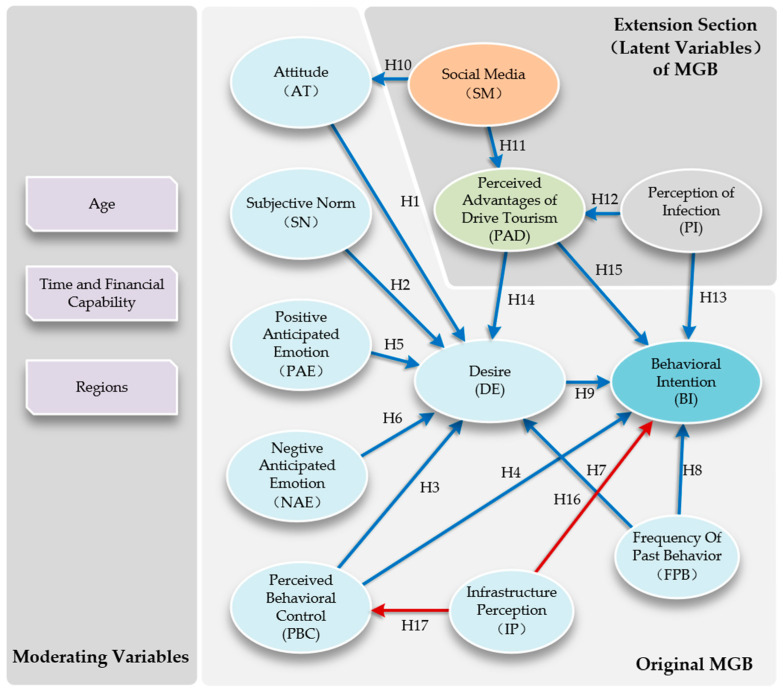
Conceptual model.

Based on the preceding discussion, we propose that attitude, subjective norms, and perceived behavioral control have a positive effect on the desire for RV tourism, as outlined in the following hypotheses (Hypotheses can be seen in Figure 1):

**Hypothesis 1** **(H_1_).** *Attitude has a positive influence on desire.*

**Hypothesis 2** **(H_2_).** *Subjective norm has a positive influence on desire.*

**Hypothesis 3** **(H_3_).** *Perceived behavioral control has a positive influence on desire.*

**Hypothesis 4** **(H_4_).** *Perceived behavioral control has a positive influence on intention.*

#### 2.3.2. Relationships Between Anticipated Emotion and Desire

Anticipated emotions are considered one of the determining factors of intention. In situations of uncertain future behavior, individuals might generate emotional expectations toward future actions [36]. These expectations can be either positive or negative, serving as motivation to encourage positive situations and avoid negative ones [26]. Both positive and negative anticipated emotions play a crucial role in forming desires.

Therefore, this paper proposes that both positive and negative anticipated emotions significantly impact desire. The hypotheses are as follows:

**Hypothesis 5** **(H_5_).** *Positive anticipated emotion has a positive influence on desire.*

**Hypothesis 6** **(H_6_).** *Negative anticipated emotion has a positive influence on desire.*

#### 2.3.3. Relationship Between Past Behavior, Desire, and Intention

The frequency of past behavior is often integrated into theoretical frameworks to explain individual decision-making processes [30]. Several studies in the field of tourism have supported the significant impact of past behavior frequency on tourists’ desires and intentions [15,32,33]. This paper proposes the following hypotheses regarding the influence of past behavior frequency on desire and intention:

**Hypothesis 7** **(H_7_).** *Past behavior frequency has a positive influence on desire.*

**Hypothesis 8** **(H_8_).** *Past behavior frequency has a positive influence on intention.*

In the MGB framework, desire mediates the relationship between antecedent variables and behavioral intention, validated across studies [15,24,32,33,37,38], showing varying correlations across contexts. Antecedents indirectly influence intention through desire [25,39], enabling comparative analysis of transformation disparities among groups. Moderating effects inform differentiated marketing to enhance tourists’ desire-to-intention transformation, as elaborated later. Therefore, the hypothesis is as follows:

**Hypothesis 9** **(H_9_).** *Desire has a positive influence on intention.*

#### 2.3.4. Perception of Intention, Social Media, Perceived Advantages of Drive Tourism, and Infrastructure Perception

In the 21st century, humanity has experienced the following five epidemic outbreaks: SARS in 2002–2003, H1N1 in 2009, Middle East Respiratory Syndrome (MERS) in 2012, Ebola in 2014, and COVID-19 starting in 2020, categorized as a pandemic [10]. COVID-19, known for its high variability and transmissibility, has brought substantial uncertainty to the future development of the tourism industry [40]. Studies using the extended model of goal-directed behavior (EMGB) investigated the impact of the 2009 H1N1 outbreak on international tourists’ behavioral intentions, suggesting that individuals’ perceptions of the pandemic indirectly affected international travel intentions through non-pharmaceutical interventions [24]. Research conducted in the United States indicated a relationship between geographic location, political ideologies, and the likelihood of travel during the COVID-19 period [9]. As of today, viruses like H1N1 and COVID-19 have become part of the seasonal flu after global pandemics, joining other flu viruses to cause the annual seasonal flu. Amidst the risks of viral infections, tourists’ behaviors exhibit contradictions, involving both avoidance and compensatory travel activities. Tourists tend to steer away from popular tourist destinations, opting for less crowded niche destinations to reduce infection risks. Camping in the city outskirts and admiring rural landscapes aligns with this inclination. As an integral part of drive tourism, acceptance of this mode of travel influences tourists’ desires and behavioral intentions toward RV tourism. The vigorous development of social media content in China has become a primary platform for information acquisition, communication, and experience sharing. Many bloggers engaged in RV tourism, drive tourism, and camping are enthusiastic about sharing scenic views, travel experiences, and lifestyles. In contemporary times, the influence of social media on the tourism industry has become a burgeoning area of study [41]. The existing research indicates that media factors such as social media and mass media influence individual travel behavioral intentions [10,15,32]. Therefore, this paper posits those individuals, upon encountering relevant content on social media platforms, experience emotional resonance, thereby fostering desires for travel and consequently generating behavioral intentions.

Drive tourism offers flexibility and freedom, catering to individualized travel needs [8,22]. Tourists on such tours often opt for destinations with natural landscapes. With the increased prevalence of household vehicles, more leisure time per capita, and the gradual improvement in transportation networks, drive tourism has become a primary form of travel among the Chinese population, holding significant prominence in the tourism market [42]. As a subset of drive tourism, RV tourism is believed to stem from the perceived advantages of drive tourism, contributing to the generation of desires and behavioral intentions among RV tourists.

Based on the perception of infection risk (PI), social media, and perceived advantages of drive tourism, the following relevant hypotheses are proposed:

**Hypothesis 10** **(H_10_).** *Social media has a positive influence on attitude.*

**Hypothesis 11** **(H_11_).** *Social media has a positive impact on the perceived advantages of drive tourism.*

**Hypothesis 12** **(H_12_).** *The perception of infection has a positive influence on the perceived advantages of drive tourism.*

**Hypothesis 13** **(H_13_).** *The perception of infection has a negative influence on behavioral intentions.*

**Hypothesis 14** **(H_14_).** *The perceived advantages of drive tourism positively influence desire.*

**Hypothesis 15** **(H_15_).** *The perceived advantages of drive tourism positively influence behavioral intention.*

The construction of RV infrastructure is closely associated with RV tourism. Adequate RV supplies, parking, and rest facilities are essential for the RV travel experience. As China’s RV tourism started relatively late, the infrastructure development remains weaker compared with developed countries in the RV industry. This could potentially have a negative impact on the intention to engage in RV tourism. To validate this possibility, infrastructure perception was incorporated into the behavioral decision-making process to explore its influence on the decision-making process of RV tourism. As established in this paper (Table 1), the stronger the perception of infrastructure, the more it indicates that respondents believe China’s RV infrastructure development is relatively weak. Such awareness might weaken the intention to travel, thus negatively affecting perceived behavioral control and behavioral intention.

**Hypothesis 16** **(H_16_).** *Infrastructure perception has a negative influence on perceived behavioral control.*

**Hypothesis 17** **(H_17_).** *Infrastructure perception has a negative influence on behavioral intention.*

### 2.4. The Moderating Effects of Time and Economy Capability, Age, and Regions

Understanding individual characteristics such as age and geographical location holds significant value in uncovering tourists’ behavioral intentions and the development of the tourism industry [43]. Wasaya et al. (2022) indicated that tourists’ varying educational levels moderate the relationship between subjective norms and behavioral intentions [16]. The study by Kovačić et al. (2020) demonstrated how tourists’ personalities and demographic characteristics impact tourism concerns and perception-based risk tourism behavior, affirming the moderating role of tourists’ nationalities in the relationship between personality and perception-based risk tourism behavior [44]. Kara and Mkwizu (2020) investigated the relationship between demographic factors and travel motivations among leisure tourists in Tanzania [45].

RV campsites are a crucial part of RV infrastructure, offering essential amenities such as parking, power, water supply, and waste disposal, catering to the fundamental needs of RV tourism. Moreover, these sites often feature safety measures and staff, ensuring security for RV tourists. Additionally, they provide services like dining, entertainment, and shopping, enhancing the overall RV tour experience [2]. Hence, RV campsites play a pivotal role in shaping the RV tourism experience. Their development and enhancement not only bolster revisit intentions among existing RV tourists but also attract more participants, fostering the growth of RV tourism. However, the distribution of RV campsites in China displays spatial disparities, with the majority concentrated in the east and north, while those better suited for western region RV tourism are scarce [42]. Therefore, grouping the sample based on the distribution density of RV campsites and conducting invariance analysis can help comprehend the impact of the development level of RV infrastructure on the decision-making process of RV tourism behavior.

Time and finances are pivotal factors in travel planning, shaping the quality and feasibility of a trip. While tourism offers individuals an opportunity for relaxation and self-exploration, it necessitates an investment of both time and money [46]. Having more leisure time does not necessarily imply the ability to afford higher financial costs, and vice versa. Integrating these two factors into Time and Economy capability allows for a more comprehensive consideration of the advantages and constraints individuals face during travel. Grouping individuals based on this Time and Economy capability aids in a more accurate understanding of the limitations and advantages individuals experience regarding time and finances. Therefore, using Time and Economy capability as a basis for grouped moderation helps comprehensively consider the combined impact of variables determined by both time and financial factors on individuals.

**Figure 2:**
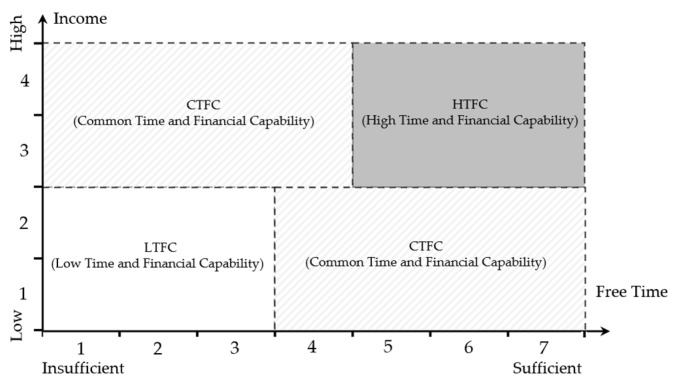
Samples grouped according to Time and Economy capability.

**Figure 3:**
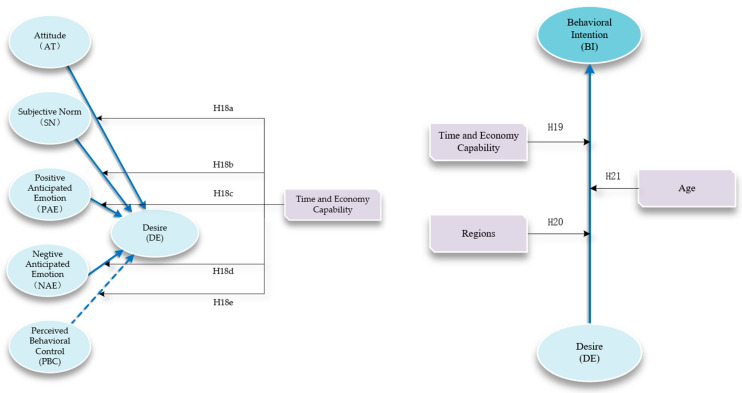
Hypotheses of the moderation effect.

The participants in this study were grouped based on age into younger and elder groups using the median age as a criterion. Additionally, as illustrated in Figure 2, they were classified based on their Time and Economy capability into three categories as follows: low Time and Economy capability (LTEC), indicating individuals with limited leisure time and lower income; common Time and Economy capability (CTEC), representing those with either more leisure time and lower income or less leisure time and higher income; and high Time and Economy capability (HTEC), denoting individuals with more leisure time and higher income. In China, drive tourism and RV camping sites are predominantly concentrated in the eastern regions, particularly the Bohai Bay Delta, Yangtze River Delta, and Pearl River Delta regions [42]. These areas were referred to as focal regions in this study, and participants were categorized into key regions (KRs) and non-key regions (NKRs). As illustrated in Figure 3, considering these grouping criteria, the study proposes the following hypotheses:

**Hypothesis 18a** **(H_18a_).** *Time and Economy capability moderates the relationship between attitudes and desire.*

**Hypothesis 18b** **(H_18b_).** *Time and Economy capability moderates the relationship between subjective norms and desire.*

**Hypothesis 18c** **(H_18c_).** *Time and Economy capability moderates the relationship between positive anticipated emotion and desire.*

**Hypothesis 18c** **(H_18d_).** *Time and Economy capability moderates the relationship between negative anticipated emotion and desire.*

**Hypothesis 18d** **(H_18e_).** *Time and Economy capability moderates the relationship between perceived behavioral control and desire.*

**Hypothesis 19** **(H_19_).** *Time and Economy capability moderates the relationship between desires and behavioral intentions in the EMGB.*

**Hypothesis 20** **(H_20_).** *Region moderates the relationship between desires and behavioral intentions in the EMGB.*

**Hypothesis 21** **(H_21_).** *Age moderates the relationship between desires and behavioral intentions in the EMGB, with older individuals being more likely to translate desires into behavioral intentions.*

## 3. Methods

### 3.1. Measurement and Definition of Variables

Through an extensive review of behavior decision-making studies based on models such as TRA, TPB, and MGB, and relevant literature on tourist behavior in the context of public health measures, RV tourism, and drive tourism, and by referencing the authoritative survey questionnaire of the MGB model, the initial questionnaire items for this study were formulated [8,16,22,24,26,27,30,31,32,34,35,47]. To ensure the effectiveness and understandability of the questionnaire items, experts and participants in the RV tourism and drive tourism industries, public health professionals, and relevant social media operators were invited to participate in trial completions and provide feedback on the proposed items related to RV tourism infrastructure, perceived advantages of drive tourism, social media, and perception of infection. The input from industry experts, participants, public health workers, and social media operators, as well as regular RV tourists, was crucial in refining and enhancing the readability, relevance, and focus of the survey questionnaire.

To ensure the authority of the questionnaire items, six attitude items, four subjective norm items, three positive anticipated emotion items, three negative anticipated emotion items, five perceived behavioral control items, four desire items, and three behavioral intention items in the questionnaire (Table 1) were referenced from previous surveys in MGB research [15,24,32], which were based on several early studies in social psychology models. The expanded components of the MGB model, including social media, the perceived advantage of drive tourism, and the perception of infection, were developed by referring to the relevant literature on social media, public health event behavioral intentions, drive tourism, and RV tourism [8,22,24,32], which were further refined by incorporating feedback from experts. Six perceived advantage of drive tourism items, four infrastructure perception items, four social media items, and eight infection risk perception items were included in the resulting questionnaire.

After formulating all items, the research team collected preliminary responses from volunteers and sought opinions on language expression. The English translation of the questionnaire items was further optimized based on feedback, addressing instances of ambiguity to ensure clarity and conciseness. The attitude, subjective norm, affective expectations, and other variables in the questionnaire employed a Likert seven-point scale, allowing participants to select from “strongly disagree” (1) to “strongly agree” (7). Additionally, past behavior frequency was measured using a seven-point scale, with respondents indicating the frequency of RV tourism from “never” (1) to “very frequently” (7).

### 3.2. Data Collections and Demographic Profile

This study employed a hybrid methodology, integrating online and on-site surveys for the collection of questionnaire data. The online survey utilized the questionnaire platform “Sojump” for questionnaire editing. Through the Static Traffic Committee of Chongqing Highway and Transportation Society, links to the questionnaire were sent via a WeChat official account to members of affiliated RV associations and online RV communities, offering incentives for participation. Concurrently, on-site visits were conducted at several RV camping sites for RV tourists in central regions in China to administer the survey, with the decision to use either paper or electronic questionnaires tailored to the individual respondents’ proficiencies with electronic devices. Out of the 547 links sent for the online survey, 363 valid responses were received. A total of 260 questionnaires were distributed in the on-site survey, and 257 were collected. The overall response rate was 76.8%. Drawing parallels from previous research on travel behavioral intentions, such as Meng and Choi’s study [15] on slow travel intentions among experienced tourists and Lee et al.’s (2012) investigation [24] into international tourism behavioral intentions, this study specifically targeted individuals from various regions in China who had prior experience in RV tourism. By employing screening questions including “Have you ever participated in RV tourism?” and “Please specify the model or type of RV you have recently traveled”, the sample was refined to encompass only those with at least one RV tourism experience. Moreover, after rigorous screening to eliminate unsuitable responses (e.g., excessively fast completion times and pattern answers), a total of 545 questionnaires were deemed suitable for further analysis. This study adhered to the principles outlined in the Declaration of Helsinki and received approval from the Institutional Review Board of the Static Traffic Committee of Chongqing Highway and Transportation Society (Approval Number: 202302, Date: 28 January 2023).

The descriptive information of the sample indicates that among the surveyed tourists, 64% were male, and 36% were female. The majority of respondents had a monthly income ranging from CNY 0 to CNY 5000 (constituting 30.2%) and CNY 5001 to CNY 9000 (constituting 51.2%). Overall, 70.6% of the respondents reported being married. Regarding educational attainment, the majority of respondents held a bachelor’s degree or higher (constituting 54.2%). The survey was conducted during the prevalence of infectious diseases, with outbreaks of influenza, COVID-19, and other infectious viruses simultaneously occurring in China and attracting public attention.

## 4. Results

### 4.1. Measurement Model

Structural equation modeling was performed through the use of AMOS24.0 and SPSS26.0 (IBM company, Chicago, IL, USA), and the resulting 545 samples were substituted for the analysis. According to the two-step approach proposed by Anderson and Gerbing (1988) [48], the fit of the model was assessed by fitting the theoretical model to the actual observed data using CFA before proceeding with the formal data analysis. If the fit was good, the second step was carried out to test causality using structural equation modeling (SEM). The CFA was divided into two main parts as follows: reliability analysis and validity analysis. The standardized loadings, Cronbach’s alpha, and composite reliability of each factor to be measured are shown in Table 1, and the average variance extracted (AVE) for each construct, the correlation coefficients, and the squared correlation coefficients between each pair of constructs are presented in Table 2. Cronbach’s alpha was between 0.818 and 0.924, both of which are greater than 0.7, and the composite reliability was between 0.601 and 0.929, indicating that the scale has good internal consistency and good reliability [49,50]. The average variance extracted (AVE) for each construct was greater than 0.5 and exceeded the squared correlation coefficients with other constructs, indicating good convergent and discriminant validity of the scale. At this point, the measurement model passed the CFA. The statistical indicators of the fit of the measurement model indicated that the model matched the data well (χ^2^ = 1597.393, df = 1120, *p* < 0.001, RESEA = 0.028, CFI = 0.967, NFI = 0.899).

### 4.2. Comparing the Models

In Table 3, the MGB model had a good fit to the data (χ^2^ = 903.534, df = 369, *p* < 0.001, RESEA = 0.052, CFI = 0.930, NFI = 0.888). In contrast, the EMGB provided richer information in explaining behavioral intentions in RV tourism, in which the R^2^ of behavioral intentions and desires was significantly higher than that of the MGB model and was able to explain more behavioral intention variations, thus predicting an individual’s behavioral desires and behavioral intentions more accurately. In addition, EMGB showed an excellent fit to the data (χ^2^ = 2351.4, df = 1208, *p* < 0.001, RESEA = 0.042, CFI = 0.922, NFI = 0.853), which was close to the minimum values of criteria suggested by Hair et al. (2009) [51], further validating its superiority relative to the MGB model. In summary, the results of this study indicate that the EMGB model is superior to the MGB model in explaining both behavioral desires and behavioral intentions in RV tourism and shows higher explanatory power and goodness-of-fit in predicting behavioral intentions.

### 4.3. Test of Hypotheses Between Latent Variables

**Figure 4:**
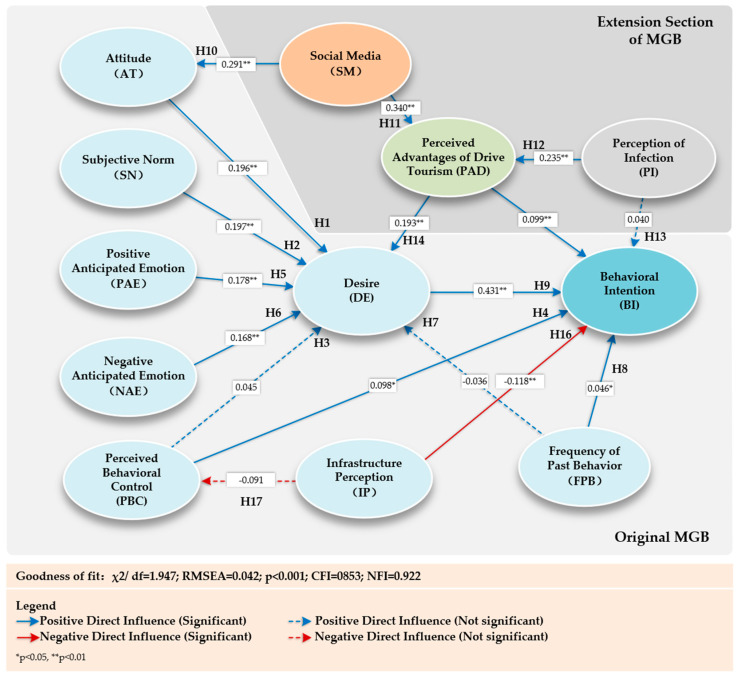
Findings from the structural equation model.

The path coefficients, significant rank, and goodness-of-fit between the variables are shown in Figure 4 and Table 4. The SEM goodness-of-fit index shows that the model in this paper is well adapted to the data. In the original MGB model, the attitude, subjective norms, positive anticipated emotion, and negative anticipated emotion predictor variables had a significant positive effect on desire (β_AT→DE_ = 0.196, t = 7.593, *p* < 0.01; β_SN→DE_ = 0.197, t = 5.538, *p* < 0.01; β_PAE→DE_ = 0.178, t = 4.921, *p* < 0.01; β_NAE→DE_ = 0.168, t = 4.690, *p* < 0.01), and hypotheses H1, H2, H5, and H6 were accepted. However, the perceived behavioral control and frequency of past behavior predictor variables did not have a significant effect on desire (β_PBC→DE_ = 0.045, t = 1.480, *p* > 0.05, not significant; β_FPB→DE_ = 0.36, t = 1.746, *p* > 0.05, not significant), and hypotheses H3 and H7 were rejected. The perceived behavioral control, past for frequency, and desire predictor variables had a significant positive effect on behavioral intentions (β_PBC→BI_ = 0.098, t = 2.985, *p* < 0.01; β_FPB→BI_ = 0.046, t = 2.109, *p* < 0.05; β_DE→BI_ = 0.431, t = 7.183, *p* < 0.01; β_NAE→DE_ = 0.168, t = 4.690, *p* < 0.01), and hypotheses H4, H8, and H9 were accepted. The infrastructure perception predictor variable had a significant negative effect on behavioral intention and a non-significant negative effect on perceived behavioral control (β_IP→PBC_ = −0.091, t = −1.755, *p* > 0.05, not significant), and hypothesis H16 was accepted and H17 was rejected.

The hypotheses proposed in this paper about the interrelationship between social media (SM), the perceived advantages of drive tourism (PAD), and the perception of infection (PI) and their effects on the behavioral decision-making process of individual RV tourism are mostly valid. The detailed results are as follows: SM and the PI had a significant positive effect on the PAD, the PAD had a significant positive effect on the DE and BI (β_SM→PAD_ = 0.340, t = 7.580, *p* < 0.01; β_PI→PAD_ = 0.235, t = 5.893, *p* < 0.01; β_PAD→DE_ = 0.193, t = 7.030, *p* < 0.01; β_PAD→BI_ = 0.099, t = 3.211, *p* < 0.05), and hypotheses H11, H12, H14, and H15 were accepted, whereas SM had a significant positive effect on the AT and BI (β_SM→AT_ = 0.291, t = 5.921, *p* < 0.01), and the PI did not have a significant effect on the BI (β_PI→BI_ = 0.040, t = 1.646, *p* > 0.05, not significant), and hypothesis H13 was rejected.

### 4.4. Multiple Group Invariance Analysis

This study conducted multiple-group invariance analyses to test the moderating effects of Time and Economy capability on the relationships between five predictor variables (AT, SN, PAE, NAE, PBC) and desire (DE), as well as the moderating effects of Time and Economy capability, age, and region on the relationships between desire (DE) and behavioral intention (BI). In this research, the sample was categorized into two age groups (older and younger), three Time and Economy capability groups (high, common, low), and two region groups (key regions and non-key regions). Age, Time and Economy capability, and region acted as moderators in the analyses. Five out of six hypothesized paths (H_18a_, H_18b_, H_18c_, H_18d_, H_19_) for the Time and Economy capability groups and the two hypotheses (H_20_, H_21_) for age and region groups were found to be significantly moderated.

By analyzing the differences in chi-square values and degrees of freedom between the three constrained models and the default model, the null hypothesis of equality between the constrained models and the default model was rejected at the *p* < 0.01 significance level. Thus, the moderation tests were significant [16,52]. This indicates significant differences in the relationships between predicting behavioral intention and the overall model across different groups in this study, supporting hypotheses H18, H19, H20, and H21. The moderating effects of different groups on the proposed relationships are presented in Table 5.

## 5. Conclusions

### 5.1. Interpretation of the Mechanism of the Impact of Infection Risk on RV Tourism Behavior Decision-Making Under Multiple Factors

The EMGB model constructed with the variables added to the MGB in this paper outperforms the original model, strongly supporting this paper’s assumptions and arguing that the theoretical expansion and deepening are justified. Thus, integrating new constructs or adapting paths can better fit the model.

The comprehensive analysis of the model results underscores the intricate interplay between various factors that shape RV tourism intentions. Desire emerges as a cornerstone latent variable, underpinned by attitude, social norms, anticipated emotions, and the perceived advantages of drive tourism. This multifaceted approach emphasizes an individual’s positive predisposition towards RV tourism, social validation, self-assurance, and the anticipation of relative experiences, all of which culminate in fostering a desire for RV travel. Notably, the perceived benefits of drive tourism not only nurture this desire but also exert a direct influence on behavioral intention, reinforcing the appeal of RV tourism as a private and flexible mode of travel. Social media, as a catalyst, amplifies this effect by disseminating experiences and information, enhancing the perceived advantages, and ultimately fostering stronger behavioral intentions towards RV tourism. Interestingly, while the perception of infection risk does not directly affect intention, it fortifies the appeal of drive tourism by promoting its perceived advantages, a testament to the role of safety concerns in shaping travel preferences during uncertain times. Conversely, infrastructure perception, when lacking, presents a direct barrier to behavioral intention, emphasizing the urgency to address infrastructure shortcomings to accommodate and encourage RV tourism. Thus, these findings underscore the complex yet interconnected nature of factors that influence RV tourism intentions, pointing to the need for tailored strategies that address both the desires and concerns of potential RV tourists.

### 5.2. Interpretation of the Moderation Effect

Groups with sufficient free time and higher economic incomes show a stronger association between the effects of attitudes on the desire to travel. Specifically, this means that positive attitudes held by individuals are more likely to translate into strong RV tourism intentions when they have more time and economic resources, whereas, in contrast, positive attitudes of groups with less time and lower economic incomes translate less into travel intentions because of resource constraints. The stronger the Time and Economy capability, the weaker the influence of subjective norms, positive anticipated emotion, and negative anticipated emotion on RV tour intentions, which indicates that individuals with more time and stronger economic capacity are less influenced by the perceptions of family members and friends around them and their personal affective expectations. This emphasizes the influence of individual income and free time on the decision-making process of RV behavior.

There are also significant differences in the influence of desires on actual behavioral intentions among individuals in different subgroups. First, the path coefficient for the influence of desire on the behavioral intention of the older group is larger than that of the younger group. This indicates that the older group attaches more importance to the freedom and comfort of travel and has more realistic conditions for action, which makes it easier for them to turn their expectations and desires for RV into action because of their rich travel experience and resources. Second, desire has a greater impact on the behavioral intention of the key region group than the non-key region group, which means that individuals living in areas with better infrastructure are more likely to translate their desires into actual behavioral intentions because they may have easier access to and enjoy the amenities and services needed for RV tours. Finally, the effect of desires on behavioral intentions was greater for the higher Time and Economy capability group than for the lower Time and Economy capability group, suggesting that individuals with more time and higher economic power are more likely to translate their desires into actual behavioral intentions, perhaps because they have more resources and conditions to pursue their desired RV tour experiences. These results provide important insights into understanding the preferences and influencing factors of different groups in RV tour intention decision-making and are also important guides for related market positioning and marketing strategies.

These results imply that different groups have different decision-making styles and behavioral intentions in the RV tourism market. Understanding the differences among these groups is important for market positioning, product design, and precision marketing. From a practical point of view, developing differentiated marketing strategies and service positioning for different groups is more likely to enhance product attractiveness and market competitiveness.

### 5.3. Suggestions

We found that tourists’ perceived risk of infection positively influences their preferences for drive tourism and intentions to engage in RV tourism, which aligns with the private nature of both drive tourism and RV tourism. This underscores the importance of providing health and hygiene services during flu seasons to maintain and enhance this characteristic, thereby retaining existing customers and creating new market opportunities. Furthermore, value-added services such as RV campsites, rest areas, and organized activities can enrich consumer experiences and foster emotional connections with RV tourism. Drive tourism enthusiasts, representing a potential market segment, can be transformed into RV consumers through targeted promotion strategies.

The positive perception of the advantages of drive tourism has a discernible impact on RV tourists’ desires and behavioral intentions, making it evident that drive tourism enthusiasts constitute a crucial potential customer segment within the RV tourism market. Consequently, intensifying the promotion and advertisement of RV tourism among drive tourism enthusiasts will undoubtedly contribute significantly to the expansion of the RV tourism market. Furthermore, the positive influence of social media on RV tourism, as evident from the operational results, underscores the power of RV tourists sharing their travel experiences on these platforms. This not only attracts more individuals to embark on RV tours but also offers invaluable advice to first-timers, thereby fostering the development and localization of RV culture. By doing so, it lures potential RV tourists to experience RV tourism firsthand, creating a virtuous circle that expands the RV touring community, enhances the scale effect, and ultimately propels growth and innovation within the RV industry. The industry should fully utilize social media platforms to encourage RV tourists to share their travel experiences, fostering a community of RV travelers and facilitating cultural exchanges and experience sharing. Precision marketing and customized services can attract more potential tourists, igniting a wave of RV tourism, expanding the market scale, and ultimately driving continuous innovation and development within the RV industry.

This study identifies the moderating role of time and economic capability in the tourism decision-making process, suggesting that differentiated strategies can more effectively meet the needs of various consumer groups. For individuals with weaker Time and Economy capabilities, their desire for RV travel is significantly influenced by subjective norms, making it challenging for such desires to form and transform into behavioral intentions. Additionally, these individuals are heavily influenced by emotional expectations. To address this, we should leverage marketing campaigns that emphasize the positive values of RV travel, such as family joy and close contact with nature, resonating with consumers’ emotional expectations and demonstrating how RV travel aligns with their lifestyle and values. By doing so, we can reduce the negative impact of subjective norms on their decision-making. Furthermore, introducing cost-effective RV travel products and short, curated itineraries tailored to their needs can stimulate the transition from desire to behavioral intention. Differentiated strategies can more effectively cater to the diverse needs of various consumer groups. For example, cost-effective and long-lasting RV products should be targeted at those with ample time but lower income, whereas high-quality, short-duration options should appeal to consumers with higher income but limited free time. The desire for RV travel among younger individuals and those from regions with inadequate infrastructure is more challenging to translate into behavioral intentions. To stimulate and facilitate this transition, we should undertake measures such as optimizing infrastructure construction, introducing RV travel products and services tailored to younger demographics, strengthening market promotion and educational guidance, and providing flexible RV rental and travel solutions. These efforts aim to ignite and accelerate the conversion of their RV travel desires into actual actions.

In conclusion, RV tourism behavior is influenced by various factors. Strategies to strengthen infrastructure, offer diverse products, and enhance social media interaction can increase consumer satisfaction and participation in RV tourism. This comprehensive approach will contribute to the sustainable growth of China’s RV tourism industry, fostering a more dynamic and competitive market.

### 5.4. Limitations and Suggestions for Future Research

This study is subject to several limitations. Firstly, because of the still limited scale of RV tourism in China, the collection of on-site questionnaires was relatively scarce, and the majority of data gathered online may have failed to encompass much older RV tourists owing to the digital divide. Future research should employ more extensive survey methods to test the practicality of this framework in RV tourism. Additionally, the lack of accurate data on China’s RV infrastructure construction and campsite inventory hindered our ability to analyze the moderating effect of regional variables on the paths. Consequently, we relied on descriptions from the existing literature and information provided by RV tourism associations to categorize key and non-key regions. While the results were significant and aligned with expectations, further research into the regional differences in factors influencing RV tourism behavioral intentions, once relevant data are released and accurately acquired, is warranted. Thirdly, as RV tourism is in its infancy in China and the market needs to be expanded, future studies on potential RV tourists, particularly drive tourism enthusiasts, and their behavioral intentions towards RV tourism can significantly contribute to market expansion, carrying substantial practical implications. Furthermore, given the notable influence of social media on the RV tourism decision-making process, as revealed by our findings, a more nuanced understanding of the differences among various social media audiences could be achieved by segmenting social media into different types for future research.

Although the extended model of goal-directed behavior is widely used in the tourism and transportation literature, its applicability to RV tourism remains relatively unexplored. By incorporating social media, perceived risk factors related to contagion, and perceived advantages of self-driving into our study, we demonstrated that the addition of specific constructs could better explain the RV tourism decision-making process, thereby enriching the contexts in which EMGB is employed. Additionally, the introduction and validation of the construct of Time and Economic Capability and its moderating effects enhanced our understanding of the differentiated behavioral intentions towards RV tourism among different groups.

## Figures and Tables

**Table 1:** Measurement items, standardized loadings, Cronbach’s alpha, and composite reliability.

Variables and Measurement Items Strongly Disagree (1)/Strongly Agree (7)	Standardized Loadings	Cronbach’s Alpha	Composite Reliability
Attitude (AT)		0.872	0.880
I think that RV tourism is positive.	0.867		
I think that RV tourism is useful.	0.745		
I think that RV tourism is valuable.	0.745		
I think that RV tourism is beneficial.	0.760		
I think that RV tourism is attractive.	0.733		
Subjective Norm (SN)		0.853	0.844
Most people who are important to me support my decision to go on RV tours.	0.877		
Most people who are important to me think it is okay for me to go on RV tours.	0.706		
Most people who are important to me want to go on RV tours with me.	0.722		
Most people who are important to me are happy to listen when I share my experiences in RV tours.	0.718		
Positive Anticipated Emotion (PAE)		0.831	0.651
If I succeed in achieving my goal (going on an RV tour), I will feel happy.	0.885		
If I succeed in achieving my goal (going on an RV tour), I will feel excited.	0.779		
If I succeed in achieving my goal (going on an RV tour), I will feel satisfied.	0.751		
Negative Anticipated Emotion (NAE)		0.818	0.601
If I fail in achieving my goal (going on an RV tour), I will feel angry.	0.862		
If I fail in achieving my goal (going on an RV tour), I will feel disappointed.	0.711		
If I fail in achieving my goal (going on an RV tour), I will feel sad.	0.745		
Perceived Behavioral Control (PBC)		0.884	0.910
I have enough time to go on an RV tour.	0.900		
I am healthy enough to go on an RV tour.	0.787		
I have enough resources (money) to go on an RV tour.	0.788		
Whether or not I travel for slow travel is completely up to me.	0.807		
I am confident that if I want, I can go on an RV tour.	0.801		
Infrastructure Perception (IP)		0.862	0.874
Although the number of RV campsites continues to increase, it there are still not enough to meet the demands of RV tour activities.	0.808		
Despite continuous improvement in the facilities at campsites, they still cannot fully meet the diverse needs of RV tourists.	0.802		
Although the installation of water and electrical hookups has increased, it still cannot fully meet the demand for a higher-quality RV tour experience.	0.774		
Although transportation to the scenic spots is convenient, the current RV tour routes are still not diverse enough.	0.799		
Social Media (SM)		0.864	0.621
I have seen content related to drive tourism, RV tourism, and camping on social media platforms.	0.859		
In recent years, there has been an increase in content related to drive tourism, RV tourism, and camping on social media platforms compared with before.	0.772		
Experiences of traveling to a certain destination based on recommendations from social media platforms.	0.754		
Social media content related to drive tourism, RV tourism, and camping has caught my interest.	0.762		
Perceived Advantages of Drive Tourism (PAD)		0.880	0.868
In recent years, I have been more eager for drive tourism than ever before.	0.845		
Recently, I have been considering drive tourism itineraries.	0.688		
When traveling, I would prioritize drive tourism and choose to drive myself.	0.709		
Drive tourism and camping as travel options can provide me with a better travel experience.	0.701		
The risk of contracting a viral infection is lower when traveling by private car.	0.686		
The risk of contracting a viral infection is higher when traveling by public transportation.	0.705		
Perception of Infection (PI)		0.924	0.929
People around me seem to refrain from travel outside because of influenza.	0.889		
Exposure outdoors can lead to infection due to influenza.	0.758		
I want to keep my distance from others because of influenza.	0.786		
Once infected, it can have more serious effects on the body.	0.790		
Being exposed outdoors increases the risk of infection due to influenza.	0.770		
I am worried about infecting family members because of my own infection.	0.769		
It is dangerous to travel outside because of influenza.	0.773		
Being infected with the influenza virus makes me feel uncomfortable.	0.756		
Desire (DE)		0.831	0.7375
I want to travel by RV in the near future.	0.659		
I wish to travel by RV in the near future.	0.619		
I look forward to travel by RV in the near future.	0.630		
I am eager to travel by RV in the near future.	0.661		
Behavioral Intention (BI)		0.844	0.827
I intend to travel by RV in the near future.	0.841		
I am planning to travel by RV in the near future.	0.735		
I will consider going on an RV tour in the near future.	0.725		
I will make an effort to travel by RV in the near future.	0.645	-	-

Note. Goodness-of-fit statistics: χ^2^ = 1597.393 (df = 1120, *p* < 0.001), RESEA = 0.028, CFI = 0.967; NFI = 0.899.

**Table 2:** Correlations among latent constructs (squared correlation).

	AT	SN	PAE	NAE	PBC	IP	DE	SM	PI	PAD	BI
AT	1										
SN	0.313 (0.098)	1									
PAE	0.277 (0.077)	0.361 (0.130)	1								
NAE	0.372 (0.138)	0.45 (0.205)	0.372 (0.138)	1							
PBC	0.164 (0.027)	0.068 (0.005)	0.136 (0.018)	0.12 (0.014)	1						
IP	−0.319 (0.102)	0.398 (0.158)	0.291 (0.085)	0.331 (0.110)	0.034 (0.001)	1					
DE	0.549 (0.301)	0.489 (0.239)	0.346 (0.120)	0.331 (0.110)	0.251 (0.063)	−0.393 (0.154)	1				
SM	0.274 (0.075)	0.335 (0.112)	0.335 (0.112)	0.402 (0.162)	0.167 (0.028)	−0.359 (0.129)	0.402 (0.162)	1			
PI	0.241 (0.058)	0.346 (0.120)	0.327 (0.107)	0.327 (0.107)	0.251 (0.063)	−0.407 (0.166)	0.398 (0.158)	0.337 (0.114)	1		
PAD	0.365 (0.133)	0.397 (0.158)	0.397 (0.158)	0.4 (0.160)	0.177 (0.031)	0.331 (0.110)	0.514 (0.264)	0.398 (0.158)	0.331 (0.110)	1	
BI	0.423 (0.179)	0.514 (0.264)	0.291 (0.085)	0.4 (0.160)	0.194 (0.038)	0.377 (0.142)	0.593 (0.352)	0.441 (0.194)	0.304 (0.092)	0.647 (0.419)	1
AVE	0.880	0.576	0.848	0.818	0.669	0.633	0.413	0.867	0.62	0.868	0.547

Note 1. The numbers in the parenthesis indicate squared correlation among latent constructs; all correlations are significant at *p* < 0.01. Note 2. AT = attitude; SN = subjective norm; PAE = positive anticipated emotion; NAE = negative anticipated emotion; PBC = perceived behavioral control; IP = infrastructure perception; DE = desire; SM = social media; PI = perception of infection; PAD= perceived advantages of drive tourism; BI = behavioral intention. Note 3. AVE = average variance extracted. Note 4. Frequency of past behavior (FPB) was not included in the measurement model since it was a single indicator.

**Table 3:** Comparison of the three models.

	χ^2^	d*f*	χ^2^/d*f*	NFI	CFI	RMSEA	R^2^ (DE)	R^2^ (BI)
MGB	903.534	369	2.449	0.888	0.930	0.052	0.42	0.36
EMGB	2351	1208	1.947	0.853	0.922	0.042	0.63	0.59
Suggested value	-	-	<3.0	>0.90	>0.90	<0.08	-	-

Note: R^2^ (DE) = R^2^ for desires; R^2^ (BI) = R^2^ for behavioral intention.

**Table 4:** The results for the EMGB.

	Direct Effect					Indirect Effect					Total Effect				
	AT	PBC	PAD	DE	BI	AT	PBC	PAD	DE	BI	AT	PBC	PAD	DE	BI
AT				0.196 **						0.084 **				0.196 **	0.085 **
SN				0.197 **						0.085 **				0.197 **	0.085 **
PAE				0.178 **						0.077 **				0.178 **	0.077 **
NAE				0.168 **						0.072 **				0.168 **	0.072 **
PBC				0.045 *	0.098 **					0.019				0.045	0.117 **
IP		−0.091			−0.118 **				−0.004	−0.011		−0.091		−0.004	−0.129 **
DE					0.431 **										0.431 **
SM	0.291 **		0.340 **						0.122 **	0.086 **	0.291 **		0.340 **	0.122 **	0.086 **
PI			0.235 **		0.040				0.045 **	0.043 **			0.235 **	0.045 **	0.082 **
PAD				0.193 **	0.099 **					0.083 **				0.193 **	0.182 **
FPB				0.036	0.046 *					0.015				0.036	0.061 *

* *p* < 0.05, ** *p* < 0.01.

**Table 5:** The results for the proposed relationships for multi-groups.

Constructs		Younger		Elder
Desire	Behavioral Intention	0.385 **		0.531 **
Constructs		KR		NKR
Desire	Behavioral Intention	0.557 **		0.223 *
Constructs		HTEC	CTEC	LTEC
Attitude	Desire	0.338 **	0.163 **	0.159 **
Subjective Norm	Desire	0.133 *	0.159 **	0.311 **
Positive Anticipated Emotion	Desire	0.121 *	0.166 **	0.200 **
Negative Anticipated Emotion	Desire	0.099	0.189 **	0.199 **
Perceived Behavioral Control	Desire	−0.006	0.091	0.047
Desire	Behavioral Intention	0.760 **	0.448 **	0.24 *

Note 1: KRs = key regions; NKRs = non-key regions. Note 2: HTEC = high Time and Economy capability; CTEC = common Time and Economy capability; LTEC = low Time and Economy capability. Note 3: * *p* < 0.05, ** *p* < 0.01.

## Data Availability

The datasets presented in this article are not readily available because the data are part of an ongoing study. Requests to access the datasets should be directed to the corresponding author of this article.

## References

[1] Dentice D., McDonald D. (2023). Trends in RV Leisure Travel amid COVID-19: Deep East Texas and Beyond. The Palgrave Handbook of Global Social Change.

[2] Caldicott R.W., Scherrer P., Harris A. (2022). The RV camping framework for understanding modern camping practices. Tour. Manag. Perspect..

[3] Ministry of Transport of the People’s Republic of China (2024). 2023 Development Statistics Report of the Transportation Industry. https://xxgk.mot.gov.cn/2020/jigou/zhghs/202406/t20240614_4142419.html.

[4] Abbas J., Mubeen R., Iorember P.T., Raza S., Mamirkulova G. (2021). Exploring the impact of COVID-19 on tourism: Transformational potential and implications for a sustainable recovery of the travel and leisure industry. Curr. Res. Behav. Sci..

[5] Lee C.-C., Chen M.-P. (2021). Ecological footprint, tourism development, and country risk: International evidence. J. Clean. Prod..

[6] Škare M., Soriano D.R., Porada-Rochoń M. (2021). Impact of COVID-19 on the travel and tourism industry. Technol. Forecast. Soc. Change.

[7] Rostami A., Kamjoo E., Bamney A., Gupta N., Savolainen P.T., Zockaie A. (2023). Investigating changes in travel behavior over time in response to the COVID-19 pandemic. Transp. Res. Part F Traffic Psychol. Behav..

[8] Fjelstul J. (2014). RV Association Members’ Profile: A Demographic Segmentation and Lifestyle Exploration. J. Tour. Insights.

[9] Vukomanovic J., Barbieri C., Knollenberg W., Yoshizumi A., Arroyo C.G. (2022). To travel or not to travel during COVID-19: The influence of political ideology on travel intentions in the USA. Ann. Tour. Res. Empir. Insights.

[10] Qiao G., Zhao X.-L., Xin L., Kim S. (2021). Concerns or desires post-pandemic: An extended MGB model for understanding South Korean residents’ perceptions and intentions to travel to China. Int. J. Environ. Res. Public Health.

[11] Liu X., Mehraliyev F., Liu C., Schuckert M. (2020). The roles of social media in tourists’ choices of travel components. Tour. Stud..

[12] Wong J.W.C., Lai I.K.W., Tao Z. (2020). Sharing memorable tourism experiences on mobile social media and how it influences further travel decisions. Curr. Issues Tour..

[13] Hardy A., Gretzel U. (2010). Why we travel this way: An exploration into the motivations of recreational vehicle users. Drive Tourism.

[14] Lee C.-K., Ahmad M.S., Petrick J.F., Park Y.-N., Park E., Kang C.-W. (2020). The roles of cultural worldview and authenticity in tourists’ decision-making process in a heritage tourism destination using a model of goal-directed behavior. J. Destin. Mark. Manag..

[15] Meng B., Choi K. (2016). The role of authenticity in forming slow tourists’ intentions: Developing an extended model of goal-directed behavior. Tour. Manag..

[16] Wasaya A., Prentice C., Hsiao A. (2022). The influence of norms on tourist behavioural intentions. J. Hosp. Tour. Manag..

[17] Hudson S. (2012). Drive tourism: Trends and emerging markets. Tour. Manag..

[18] Rogerson C.M., Rogerson J.M. (2021). Mundane urban tourism: The historical evolution of caravan parks in South Africa 1930–1994. Urban Tourism in the Global South: South African Perspectives.

[19] Pearce P.L., Wu M.Y. (2018). A mobile narrative community: Communication among senior recreational vehicle travellers. Tour. Stud..

[20] Shin Y.H., Severt K., Fjelstul J. (2017). RV traveler’s pull factors to campgrounds in leisure tourism. J. Qual. Assur. Hosp. Tour..

[21] Fodness D., Murray B. (1997). Tourist information search. Ann. Tour. Res..

[22] Fjelstul J., Wang Y., Li X. (2012). Examining the RV travelers’ camping experience: A social media approach. Tour. Anal..

[23] Lumsdon L.M., Page S.J. (2007). Tourism and Transport.

[24] Lee C.-K., Song H.-J., Bendle L.J., Kim M.-J., Han H. (2012). The impact of non-pharmaceutical interventions for 2009 H1N1 influenza on travel intentions: A model of goal-directed behavior. Tour. Manag..

[25] Bagozzi R.P., Warshaw P.R. (1992). An examination of the etiology of the attitude-behavior relation for goal-directed behaviors. Multivar. Behav. Res..

[26] Perugini M., Bagozzi R.P. (2001). The role of desires and anticipated emotions in goal-directed behaviours: Broadening and deepening the theory of planned behaviour. Br. J. Soc. Psychol..

[27] Ajzen I. (1985). From intentions to actions: A theory of planned behavior. Action Control.

[28] Zhao P., Gao Y. (2022). Public transit travel choice in the post COVID-19 pandemic era: An application of the extended Theory of Planned behavior. Travel Behav. Soc..

[29] Morten A., Gatersleben B., Jessop D.C. (2018). Staying grounded? Applying the theory of planned behaviour to explore motivations to reduce air travel. Transp. Res. Part F Traffic Psychol. Behav..

[30] Conner M., Armitage C.J. (1998). Extending the theory of planned behavior: A review and avenues for further research. J. Appl. Soc. Psychol..

[31] Taylor S.A. (2007). The addition of anticipated regret to attitudinally based, goal-directed models of information search behaviours under conditions of uncertainty and risk. Br. J. Soc. Psychol..

[32] Bui N.A., Kiatkawsin K. (2020). Examining Vietnamese Hard-Adventure Tourists’ Visit Intention Using an Extended Model of Goal-Directed Behavior. Sustainability.

[33] Chen H., Wang L., Xu S., Law R., Zhang M. (2022). Research on the Influence Mechanism of Intention to Proximity Travel under the COVID-19. Behav. Sci..

[34] Ajzen I. (1980). Understanding Attitudes and Predicting Social Behavior.

[35] Ajzen I. (2002). Perceived Behavioural Control, Self-efficacy, Locus of Control and the Theory of Planned Behaviour. J. Appl. Soc. Psychol..

[36] Gleicher F., Boninger D.S., Strathman A., Armor D., Hetts J., Ahn M. (1995). With an eye toward the future: The impact of counterfactual thinking on affect, attitudes, and behavior. What Might Have Been: The Social Psychology of Counterfactual Thinking.

[37] Lanzini P., Khan S.A. (2017). Shedding light on the psychological and behavioral determinants of travel mode choice: A meta-analysis. Transp. Res. Part F Traffic Psychol. Behav..

[38] Bae J.-S., Chiu W., Nam S.-B. (2024). The Model of Goal-Directed Behavior in Sports Participation: A Meta-Analysis Comparing Pre-and Post-COVID-19 Eras in the Republic of Korea. Behav. Sci..

[39] Bagozzi R.P., Dholakia U.M. (2006). Antecedents and purchase consequences of customer participation in small group brand communities. Int. J. Res. Mark..

[40] Hidalgo A., Martín-Barroso D., Nunez-Serrano J.A., Turrión J., Velázquez F.J. (2022). Does hotel management matter to overcoming the COVID-19 crisis? The Spanish case. Tour. Manag..

[41] Alghizzawi M., Salloum S.A., Habes M. (2018). The role of social media in tourism marketing in Jordan. Int. J. Inf. Technol. Lang. Stud..

[42] Ding H., Wang J., Liao W., Liang T., Dai H. (2021). Site selection of self-driving and recreational vehicle camps in China: An investigation using analytic hierarchy process and entropy. J. Traffic Transp. Eng. (Engl. Ed.).

[43] Pearce J., Moscardo G. Social representations of tourist selfies: New challenges for sustainable tourism. Proceedings of the BEST EN Think Tank XV.

[44] Kovačić S., Mărgărint M.C., Ionce R., Miljković Đ. (2020). What are the factors affecting tourist behavior based on the perception of risk? Romanian and Serbian tourists’ perspective in the aftermath of the recent floods and wildfires in Greece. Sustainability.

[45] Kara N.S., Mkwizu K.H. (2020). Demographic factors and travel motivation among leisure tourists in Tanzania. Int. Hosp. Rev..

[46] Crompton J.L. (1979). Motivations for pleasure vacation. Ann. Tour. Res..

[47] Batool T., Ross V., Brijs K., Neven A., Smeets C.J., Scherrenberg M., Dendale P., Vanrompay Y., Janssens D., Wets G. (2022). It’s how you say it–The extended Theory of Planned Behaviour explains active transport use in cardiac patients depending on the type of self-report in a hypothesis-generating study. Transp. Res. Part F Traffic Psychol. Behav..

[48] Anderson J.C., Gerbing D.W. (1988). Structural equation modeling in practice: A review and recommended two-step approach. Psychol. Bull..

[49] Bagozzi R.P., Yi Y. (2012). Specification, evaluation, and interpretation of structural equation models. J. Acad. Mark. Sci..

[50] Nunnally J.C., Bernstein I.H. (1994). Psychometric Theory.

[51] Hair J.F., Black W.C., Babin B.J., Anderson R.E. (2009). Multivariate Data Analysis.

[52] Steenkamp J.-B.E., Baumgartner H. (1998). Assessing measurement invariance in cross-national consumer research. J. Consum. Res..

